# Withholding and withdrawing in real life

**Published:** 2019-01-20

**Authors:** O Piazza

**Affiliations:** 1 Università degli Studi di Salerno (Italy)

**Keywords:** futility, ICU, mechanical ventilation

With an average of one patient deceased every six hospitalizations, in my 25 years as an intensive care doctor I have been often forced to face death. Many times, I found myself deciding whether and when it was appropriate withholding or withdrawing life-support treatments when they proved futile. As any Intensive Care physician or nurse, I was trained in the use of extracorporeal prosthetic techniques, which replace vital functions. In ICU we continually use mechanical ventilation, extracorporeal circulation systems, dialysis, and many, many drugs. Death can be postponed to a limit that was unimaginable a few decades ago. During my years as a resident in anaesthesia and intensive care, I absorbed an alternative way of seeing the functionality of the organs. Working shifts in intensive care are very long; doctors in training and their mentors live in strict contact, in a closed system that is constituted as a whole world of experiences, full of objects that are not part of everyday life. This “unnatural” condition, the availability of advanced technologies that prolong life and the contemporary and frequent contact with death, make it very difficult an open and conscious discussion of end-of-life decisions with other doctors and with relatives. I am sure that the greatest difficulty I have encountered in my work is the impossibility of speaking or communicating in any way with my patients, to be able to cure them according to their free choices on the therapy to be undertaken. Unfortunately, the vast majority of patients who die in ICU are not able to express their wishes in relation to invasive care. In Italy, the so-called “living will” or “early declarations of treatment” is not a widespread practice nor is it an imperative for the doctor who treats a patient who is not able to express his consent or his will. The ICU doctor, young and inexperienced or full of experience and responsibility, must try to “guess” the will of the person who cares and, to apply those choices that she or he would have shared if they were able to express their will, can only address his/her family. In Italy, relatives do not exercise any legal right to decide in place of the patient but are considered witnesses of the patient’s will. With Mr. Antonio’s daughter, this kind of approach did not work, to the point that two Carabinieri showed up on Friday night to check that all of us, doctors and nurses, were not abusing our power over life or death. Mr. Antonio, immobilized in his bed with a decidedly poor prognosis for more than a year, had already been the object of discussion, sometimes compassionate, sometimes cynical, among my colleagues. It was necessary to make a complex decision, the definitive one, on the suspension and non-implementation of therapies, which were considered futile for Mr. Antonio. A therapy is futile when it slows down rather than interrupting the pathological process that leads to death. It is a treatment that has no chance of therapeutic success. A futile therapy should never be undertaken, but, as often happens, orotracheal intubation and mechanical ventilation had been imposed to Antonio, to all effects a terminally ill patient. Perhaps, his prognosis had not been clearly defined in emergency conditions and perhaps not all doctors would have been able to define, so, “right now”, Antonio a “terminally ill”, one arrived at the terminus of diseases for which no effective treatment exists. Antonio is a terminal patient not because he has a tumour but because, already suffering from a chronic illness that prevented him from speaking, eating, moving and recognizing his wife, he developed a condition of multiorgan insufficiency for which our care prolonged the agony without resolving the cause of his disease for about a month, needlessly, futilely. Once the prognostic judgment has been formulated, also in relation to the results obtained in the first days of intensive care, clinicians can communicate their impressions to the family. An overall judgment should be drafted by a multidisciplinary team, involving the nursing staff in the decision and communication. In the case of Antonio and his daughter, unfortunately, the decision to suspend the intubation and ventilation, once these had proved decidedly inappropriate, was communicated by a single doctor, my most human and generous colleague, but also the youngest doctor. Antonio’s eldest daughter, with her load of uncertainty, pain and guilt, did not find any other remedy to her regret that call the police. The suspension of mechanical ventilation is followed by the patient’s death within a few hours: the colleague on duty, Friday night, re-intubated Antonio, admitting that the family had not been sufficiently prepared and that had not shared the even clear explanations already received.

-If my father bothers you, tell me where I have to move him.
-Get out of here, you offend me. I replied angrily.

I have thought so much in these days of Antonio and his daughter, and of my mother and her peaceful death, without excess, without strangers, but certainly not without pain.

“Two beautiful things has the world:/Love and Death”, wrote Leopardi^1^ for me and for Antonio and his daughter, even though he never crossed the threshold of an ICU.

I apologized, she was sorry but did not change her mind. I will be back in the ward on Monday.
